# Modeling the Quality of Player Passing Decisions in Australian Rules Football Relative to Risk, Reward, and Commitment

**DOI:** 10.3389/fpsyg.2019.01777

**Published:** 2019-08-02

**Authors:** Bartholomew Spencer, Karl Jackson, Timothy Bedin, Sam Robertson

**Affiliations:** ^1^Institute for Health & Sport, Victoria University, Melbourne, VIC, Australia; ^2^Champion Data, Pty Ltd., Melbourne, VIC, Australia

**Keywords:** motion models, spatiotemporal, decision-making, team sports, Australian Rules football, player tracking

## Abstract

The value of player decisions has typically been measured by changes in possession expectations, rather than relative to the value of a player’s alternative options. This study presents a mathematical approach to the measurement of passing decisions of Australian Rules footballers that considers the risk and reward of passing options. A new method for quantifying a player’s spatial influence is demonstrated through a process called commitment modeling, in which the bounds and density of a player’s motion model are fit on empirical commitment to contests, producing a continuous representation of a team’s spatial ownership. This process involves combining the probability density functions of contests that a player committed to, and those they did not. Spatiotemporal player tracking data was collected for AFL matches played at a single stadium in the 2017 and 2018 seasons. It was discovered that the probability of a player committing to a contest decreases as a function of their velocity and of the ball’s time-to-point. Furthermore, the peak density of player commitment probabilities is at a greater distance in front of a player the faster they are moving, while their ability to participate in contests requiring re-orientation diminishes at higher velocities. Analysis of passing decisions revealed that, for passes resulting in a mark, opposition pressure is bimodal, with peaks at spatial dominance equivalent to no pressure and to a one-on-one contest. Density of passing distance peaks at 17.3 m, marginally longer than the minimum distance of a legal mark (15 m). Conversely, the model presented in this study identifies long-range options as have higher associated decision-making values, however a lack of passes in these ranges may be indicative of differing tactical behavior or a difficulty in identifying long-range options.

## Introduction

Team sport athletes are consistently presented with situations in which their decisions effect the immediate state of a game. These consist of overt on-ball decisions relating to passing or shooting, however also include off-ball actions such as occupation of a given space. Whilst previous works have quantified the impact of a decision on some measure of possession expectation (Cervone et al., 2014, 2016; Jackson, 2016) or on measures of spatial control (Fernandez and Bornn, 2018), their value has typically been measured by the change in some metric or relative to a contextual mean. We believe the value of a player’s decision should be quantified relative to the alternative options that were available. Although a pass may yield a positive increase in a team’s scoring chance by *x*, the decision is by definition sub-optimal if alternatives exist that increase it by greater than *x*. By measuring a player’s decision relative to their options, we can quantitatively attribute value to a player’s decision-making abilities, further decoupling components of a player’s performance.

The expected possession value (EPV) metric considers spatiotemporal data, match phase and player behaviors to quantify possession outcomes in basketball (Cervone et al., 2014, 2016). Computing the change in EPV between possessions assigns a value to player possession contributions. A player’s decision is valued relative to the tendencies of other players in the same situation, producing a player’s EPVA (EPV-added over replacement) as the sum of a player’s EPV-added (*EPV_end_* – *EPV_start_*) across all possessions. In Jackson (2016), Australian Rules footballers ranking points are the sum of their possession contributions, valued relative to the event and location, an extension of the measure of field equity developed in O’Shaughnessy (2006). Similar to Cervone et al. (2014), player contributions are measured relative to mean outcomes and a player is deemed to be a good decision maker if their involvement improved their team’s field equity, a measurement of scoring chance relative to match phase and possession location. In Horton et al. (2015), football passes were labeled qualitatively using machine learning algorithms with quantitative inputs, learnt from manual labeling of passing quality by sporting professionals. The inclusion of player dominant regions, a method of bounding a player’s spatial ownership via consideration of player momentum, suggests the quality of a pass has some dependence on a team’s spatial control.

Common amongst these studies is the valuation of player decisions with respect to some change in possession expectation. Another approach would be to value decisions relative to alternative options, however, modeling this problem presents unique challenges. While quantifying a decision after the fact can be done by measuring the change in a given objective, each option available to a player has an accompanying probability of success. Multiple studies have measured the risk of passes in football. In Szczepański and McHale (2016), the success of a pass depended upon the skill of a player and their teammates, field position of the pass location and destination, and pressure. The latter was approximated dependent on a player’s typical playing positions and time between passes, rather than consideration of opponent locations due to an absence of player tracking data. Power et al. (2017) measured the risk and reward of passing options using spatiotemporal tracking data, where the risk of a pass considers player velocity, defender proximity and momentum, and possession statistics and the reward of a pass is the probability that the pass will result in a shot on goal. From their measure of risk, the risk tendencies and completion rates of players were analyzed. Our recent work in AFL produced measures of risk and reward via discrete player motion models and measures of future possession expectations respectively (Spencer et al., 2018). Passing networks have been used to describe the passing behaviors of athletes (e.g., Fewell et al., 2012; Pena and Touchette, 2012), but have not included quantitative measurements of the quality of links in these networks.

In this study we value a player’s passing decisions through consideration of the risk and reward of their options. We measure the risk of a pass through modeling of individual and team spatial control, and reward via a measure of field equity detailed in Jackson (2016). We present a new method for modeling spatial control via probabilistic modeling of player commitment to contests with consideration of their momentum. This process, referred to as commitment modeling, produces player motion models that more realistically represent player behavior based on their proximity to important events. We use the resultant decision-making model to analyze characteristics of player decision-making, its predictability, and distributions of risk taking within teams.

### Related Work

#### Motion Models

There exist many methods for representing a player’s spatial occupancy. One common approach, particularly in football, is that of Voronoi tessellations which bound a player’s owned space as the space in which they could occupy before any other player. Simple applications of this approach do not consider player orientation, velocity, or individual physical capabilities (e.g., Fonseca et al., 2012). Taki and Hasegawa (2000) produced variations incorporating a player’s orientation, velocity, but assumed consistent acceleration. Fujimura and Sugihara (2005) proposed an alternative motion equation, adding a resistive force that decreases velocity. This approach involved a generalized formula that more realistically represented a player’s inability to cover negative space if moving at speed. Gudmundsson and Wolle (2014) individualized these models, fitting a player’s dominant region from observed tracking data.

Underlying these models is an assumption that spatial ownership is binary. That is, each location on the field is owned completely by a single player, determined by the time it would take them to reach said location, henceforth referred to as their time-to-point. Through observations of contests, we propose that ownership of space is continuous. For a given location, if the time-to-point of the ball is greater than the time-to-point of at least two players, then no single player owns the space completely. This distinction is important if we wish to quantify spatial occupancy (and its creation) relative to the ball, given its time-to-point, as we need to account for changes in field formations that could occur between possessions.

Recent papers have addressed this. The density of playing groups was explored with Gaussian mixture models in Spencer et al. (2017). Spencer et al. (2018) produced a smoothed representation of a team’s control using non-probabilistic player motion models fit on observed tracking data. While a team’s ownership was expressed on a continuous scale, the use of motion models with discrete bounds may result in unrealistic estimations of a player’s influence (Brefeld et al., 2018). Fernandez and Bornn (2018) measured a player’s influence area using bivariate normal distributions that considered a player’s location, velocity, and distance to the ball. The result is a smoothed surface of control in which a team’s influence over a region is continuous, however the size of a player’s influence is within a selected range, rather than learnt from observed movements. Recently, Brefeld et al. (2018) fit player motion models on the distribution of observed player movements, utilizing these probabilistic models to produce more realistic Voronoi-like regions of control. In the interest of computing time, two-dimensional models were produced for different speed and time bands, hence the resultant models are not continuous in all dimensions.

Given its contested and dynamic nature, a continuous representation of space control is preferable (e.g., Fernandez and Bornn, 2018; Spencer et al., 2018). Furthermore, a player logically exhibits greater control over space in which they are closer, hence we develop probabilistic motion models in this paper. When probabilistic models are fit on the entirety of a player’s movements (as in Brefeld et al., 2018), we find that the probability of player reorientation is underestimated. In decision-making modeling, our interest is in measuring the contest of space that would occur if the ball were kicked to said space. Hence to represent this realistically, it is important to fit the distribution of player movements observed under similar circumstances. We model a player’s behavior when within proximity of contests. We achieve this via a procedure we call *commitment modeling*, where we fit the distribution of player commitment to contests in four dimensions (velocity, time, and x- and y- field position). The result is a realistic representation of player behaviors when presented with the opportunity to participate in a contest.

## Materials and Methods

### Data and Pre-processing

Spatiotemporal player tracking data was collected from the 2017 and 2018 AFL seasons. Data were collected by local positioning system (LPS) wearable Catapult Clearsky devices (Catapult Sports, Melbourne, VIC, Australia), situated in a pouch positioned between the players’ shoulder blades. Positional data in the form of Cartesian coordinates was recorded at a frequency of 10 Hz for all 44 players. To ensure consistent tracking and field dimensions, analyzed matches were limited to those played at Docklands Stadium, Melbourne, VIC, Australia. Play-by-play transactional data (i.e., match events such as kicks, marks, and spoils, and their associated meta-data) were manually collected by Champion Data (Champion Data, Pty Ltd., Melbourne, VIC, Australia). These events are henceforth referred to as transactions. Consolidation of transaction and tracking data was used to infer ball position from possession, as ball tracking data is not available in Australian Rules football. Datasets were joined via unix timestamps present in both datasets. Transactions were recorded to the nearest second, hence it was assumed they occurred at the beginning of a second when matched to 10 Hz tracking data. If the location of one or more players was lost during a passage of play, said passage was omitted from the analysis. In total, data from 60 matches was used in this study. A total of 2236 passes across 60 matches were analyzed in this study.

A player’s velocity and displacement direction were calculated from raw positional data. Displacement direction was extracted from consecutive tracking samples (i.e., a player’s displacement direction was recorded as the angle formed by consecutive tracking samples, relative to the positive y-axis). A player’s change in displacement direction was considered as the angle between two vectors, $\vec{AB}$ and $\vec{BC}$, where A, B, and C are the player’s three most recent positions, and the angle describes the change in displacement direction between positions B and C (Eq. 1). The same process was used to calculate the location of an event relative to a player (where A and B are a player’s previous and current position, and C is the location of interest). Velocity, recorded in meters/second, was calculated as the Euclidean distance between a player’s current position and their position, 1 s prior.

(1) $$\theta =\cos^{-1}(\frac{\vec{AB}\cdot \vec{BC}}{\left||\vec{AB}||\cdot ||\vec{BC}|\right|})$$

In this study, only player decisions following a mark were included, given that a mark provides the player with time to make an informed decision. In Australian Rules football, a mark is a kick greater than 15 m that is received by a player on the full (i.e., without bouncing). To locate the destination of a player’s kick following their mark, the next transaction must also be a mark. If the next possession following a kick is not a mark, we are unable to reliably locate the intended target, given a reliance on transactions to infer ball position.

### Commitment Modeling

For analysis purposes, a contest was defined as a transaction following a pass in which at least one player from each team was involved and the ball location (for both the preceding kick and the receive) could be inferred from the consolidated datasets. In this study, the contest transaction types were spoils and contested marks. The former is an attempted pass that was physically prevented by the opposition and the latter is a mark in which multiple players attempted to receive the ball. For each contest, interest related to two moments – the pass that preceded the contest and the contest itself. For each moment, the time (*t_p_* and *t_c_* respectively) and field formation (position, displacement direction, and velocity of all on-field players) were recorded. A player was considered as having committed to a contest if their Euclidean distance from the location of the contest was less than 2 m at *t_c_*. Using a player’s position at *t_p_* and their commitment (recorded as a binary value), a model was developed that quantified the probability a player would commit to a contest across a continuous space within their vicinity.

For each contest, we record player’s velocity, displacement direction, and position, and define the time between *t_p_* and *t_c_* as the ball’s time-to-point. For each player, compute the relative location of the contest to player displacement direction and position. If the Euclidean distance between said player’s position at time *t_c_* and the contest location is ≤ 2 m, set their commitment to 1, else commitment is set to 0 if the distance is > 2 m. A player’s velocity, commitment, the ball’s time-to-point, and the relative x- and y- co-ordinates of the contest are recorded. Given that options are only considered in a 60 m radius of the kicker, the maximum repositioning time available to a player never exceeds 4 s, hence it is unlikely that a player can relocate more than 30 m in this period. In the interest of computation time, player commitment behavior is only recorded for players within 35 m of the contest locations.

The data was separated by the binary commitment variable, and kernel density estimation (KDE) used to estimate their probability density functions (PDFs). KDE is a form of data smoothing in which the PDF of a dataset is estimated, the form of which depends on the chosen kernel function and bandwidth inputs (Silverman, 1986). KDE has previously been used in motion model studies by Brefeld et al. (2018) who produced motion models on the distribution of a player’s observed movements, regardless of context. In this study Gaussian kernel functions were used and bandwidth was set to 1.5, chosen after experimentation of different values. Datasets were four-dimensional, containing player velocity (m/s), ball time-to-point (s), and the relative x- and y- co-ordinate of the contest (m).

**FIGURE 1:**
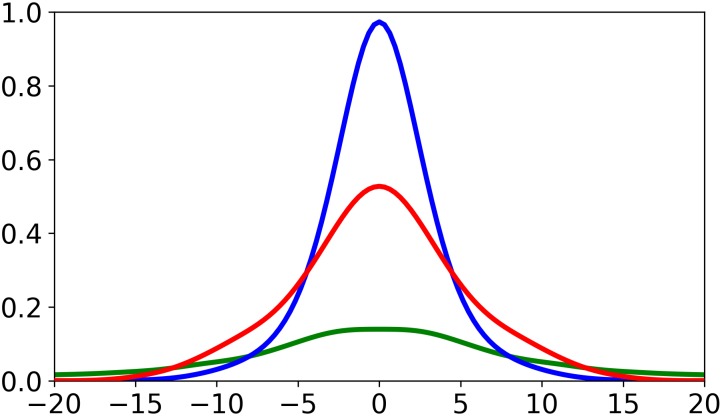
Two-dimensional representation of the commitment modeling process. The blue line represents the distribution for commitment values of 1 (*f*_*c*=1_), and the green line represents the distribution for commitment values of 0 (*f*_*c*=0_). The red line represents player influence (*P_r_(x)*), derived from the combined commitment distributions (see Eq. 2). This exemplar represents a player’s commitment probability across relative x- co-ordinates for a y- displacement of 1 m, velocity = 4 m/s, and time = 2 s. All co-ordinates are relative to player displacement direction.

Individually, these distributions represent the density of the data-sets in four dimensions. If a player’s positional information and the ball location is known, the probability they will commit to a contest at location *x* is as follows:

(2) $$\mathrm{Pr}(x)=\frac{wf_{c=1}}{wf_{c=1}+(1-w)f_{c=0}}$$

where *w* is a weighting factor equal to the size of the commitment dataset divided by the total number of samples, and *f*_*c*=1_ and *f*_*c*=0_ are the PDFs for the datasets where commitment = 1 and commitment = 0 respectively. A player’s commitment probability (Pr(*x*)) considers their position relative to *x*, their velocity, and the ball’s time-to-point. Ball time-to-point to a location is equal to the distance between the ball and the location, divided by ball velocity. Ball velocity was estimated as 18.5 m/s after manually timing kicks from two quarters of a single AFL match and taking the average, however we note that this is a rough estimation as distances were estimated from manually recorded transactions. This represents a novel method for combining the distributions of two datasets of unequal sample size, where the resulting metric quantifies the probability that a new point belongs to each distribution. The combination of these distributions in a 2D space is illustrated in Figure 1. The resultant distributions can be calculated for a player’s position, providing a distribution of the likelihood of their repositioning to each location, such that we derive a representation of their spatial influence comparable to that of traditional motion models.

### Decision-Making Model

Following a pass, the ball can be received on the full, resulting in a mark, or can be received after a bounce, in which case a mark is not awarded. Hence, each of a player’s passing options has four possible outcomes – successful passes in which a teammate receives the ball before (*A*) or after (*B*) it bounces, and unsuccessful passes in which an opponent does the same (*C* and *D* respectively). For each option, we calculate the probability (*p*) and value (*e*) of each event (Eq. 3). As we consider players to be moving objects who exhibit spatial influence over locations not at their present position, the player with the ball could theoretically kick to any location within a radius equal to their maximum kicking distance. The typical maximum range of elite footballers has been found to be between 55 and 63 m (Ball, 2008c), hence the kicking radius in this study is set to 60 m. While some locations are likely sub-optimal choices, we calculate the expected outcome (EO) of each location within said radius. The EO for a location, *x*, is as follows:

(3) $$EO(x)=p_{A}(x)e_{a}(x)+p_{B}(x)e_{a}(x)-p_{C}(x)e_{o}(x)-p_{D}(x)e_{o}(x)$$

where *e_a_* and *e_o_* are the field equity values for the attacking team and their opponent respectively. Derivation of field equity in AFL has been the focus of previous studies (O’Shaughnessy, 2006; Jackson, 2016).

From the EO of a pass, we calculate the value of a decision (referred to as the decision value or DV) as the EO of the pass that was executed, divided by the maximum EO contained in a player’s kicking range (EO_*opt*_):

(4) $$DV(x)=\frac{EO(x)}{EO_{opt}}$$

The EO of a pass will be negative if the equity at its target location is negative. For a decision with negative EO, the associated DV will likewise be negative. For a DV < -1, we set DV to -1.

#### Outcome Probabilities

For a given location, a team’s spatial influence (INF) is the sum of the influence of its players:

(5) $$INF(x)=\sum_{i=1}^{18}\underset{i}{\mathrm{Pr}}(x)$$

where Pr_*i*_ is the commitment probability array for player *i*, from Eq. 2. An attacking team’s influence is a measure of the commitment of its players. From the influence of each team, we calculate the attacking team’s spatial dominance (DOM) as:

(6) $$DOM_{a}(x)=\frac{INF_{a}(x)}{INF_{a}(x)+INF_{o}(x)}$$

where *INF_a_(x)* and *INF_o_(x)* are the influence of the attacking team and their opponent at *x*.

The attacking team’s dominance at *x* is the proportion of space they own. Logically, greater spatial dominance translates to a higher chance of a successful pass. Given that dominance is a relative measure, it is possible for a team to have high dominance over a location where influence is low. In such a case, while the probability of a successful pass is high due to their dominance, the probability that their players will reach the location is low, hence such a location is likely a poor passing location. To account for this, we calculate the probability of a successful mark (*p_A_* and *p_C_* from Eq. 3) as a team’s dominance multiplied by their influence.

(7) p(x)=DOM(x)×INF(x)

Given that a team’s desired outcome is a successful pass resulting in a mark, this probability (Eq. 7) is of particular importance when analyzing a pass. We refer to *p_A_* as the *risk* of a pass, where higher values indicate a safer passing option.

If a pass does not result in a mark, the probability that either team would win the ball is simply equal to their dominance (*p_B_* and *p_D_* from Eq. 3).

#### Kicking Variance

Given imperfect accuracy of kicks, there is a chance that a kick will not reach its intended target. To incorporate this variance, we represent the likely target of a kick using a 2D Gaussian distribution with covariance equal to 5% of the kicking distance. The modified EO of a kick is equal to the summed product of the kicking Gaussian’s PDF and the raw EO values contained in its radius:

(8) $$EO_{mod}(x)=\sum_{i\in S}EO(i)f(i)$$

where *S* is the set of integer co-ordinates in a radius around *x* equal to 5% of the Euclidean distance between the ball and *x*.

### Statistical Analysis

For each analyzed event, the optimal pass is identified as the pass to a teammate within a 60 m radius of the kicker whose EO is highest. The characteristics of the pass that was made and the pass identified as being optimal were extracted for all kicks that were preceded and resulted in a mark across the analyzed matches (see Table 1 for a list of variables and definitions). We refer to the pass that was made as the *decision* and the pass identified as the optimal option as the *alternative* (note that if the decision was optimal it will be equal to the alternative). Descriptive statistics (mean ± SD) were produced for all metrics. Spearman’s correlation coefficient (ρ) was used to measure the correlation of decision-making metrics with location. KDE was used to fit the distribution of analyzed variables, finding that the decision-making metrics are not normally distributed. The Mann–Whitney *U*-test was used to assess differences between the characteristics of decisions and alternatives (Mann and Whitney, 1947).

**Table 1:** Definitions of decision-making variables.

Variable	Definition
Dominance	The proportion of space owned by a team (see Eq. 6)
Influence	A measure of spatial occupancy irrespective of opposition locations, equal to the summed commitment probabilities of a team’s players (see Eq. 5)
Risk	The likelihood of a successful pass resulting in a mark (see Eq. 7)
Decision value (DV)	The value of a player’s passing decision, measured relative to the optimal decision available at the time of the pass (see Eq. 4)
Expected outcome (EO)	A numerical value describing the expected value of passing to a field position that considers the risk and reward of said pass (see Eq. 3)
Distance	The Euclidean distance between two points. For a kick, distance is the Euclidean distance between the location of the kicker and of the receiver


We explore team level trends in decision-making by comparing two teams. Teams were selected by taking the teams with the highest samples who fit the following criteria – one team who finished in the top 8 (*Team A*) in both the 2017 and 2018 regular AFL playing seasons, and one team who finished in the bottom 10 in the same seasons (*Team B*). Participation in the play-off finals in AFL is between the top 8 teams, hence the choice of cut-off criteria ensured one team who participated in the finals, and one non-finalist team. Furthermore, the distribution of team samples is heavily skewed, hence importance was placed on selecting teams with adequate sample sizes. This skew in team samples is due to this study’s focus on matches played at a single stadium, hence teams who played more matches at this stadium appear more frequently in the dataset. Differences between team-level statistics were measured using the Mann–Whitney *U*-test. Within-team decision-making is analyzed for both teams. We fit the distribution of mean decision-making characteristics for each player on the team. All analyses were carried out in the Python programing language, using SciPy (Jones et al., 2014) and the Scikit-learn (Pedregosa et al., 2011) packages.

## Results

### Motion Models

**FIGURE 2:**
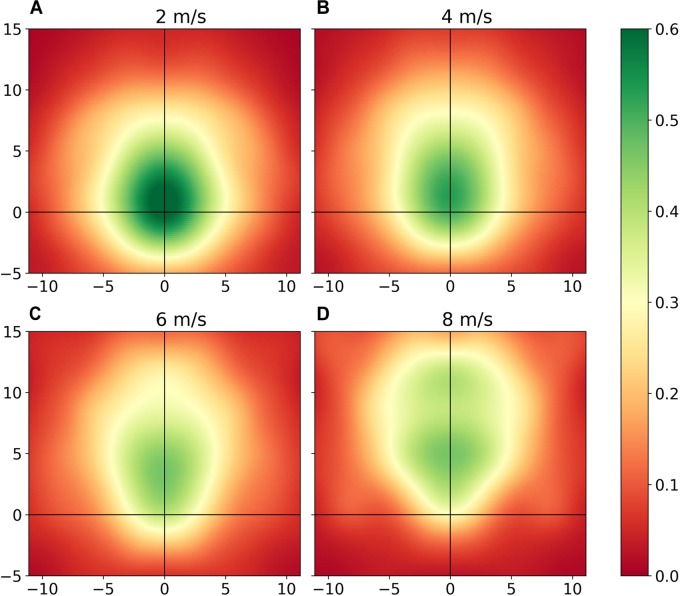
Motion models representing a player’s area of influence when moving at **(A)** 2 m/s, **(B)** 4 m/s, **(C)** 6 m/s, and **(D)** 8 m/s for ball time-to-point of 2 s. Heatmap intensity is equivalent to the probability that a player (at the point of origin) would participate in a contest at relative x-, y-co-ordinates, as quantified by observed commitment behaviors.

Motion models were produced from 46220 instances of player commitment. Within the dataset there were 6392 instances of player commitment (Commitment = 1), and 39828 instances of no commitment (Commitment = 0), producing a weighting coefficient (*w*) of 0.14. Resultant motion models for four different player velocities for ball time-to-point of 2 s are visualized in Figure 2. Peak commitment probabilities occurred at 0.8 m for a velocity of 2 m/s (Figure 2A), 1.6 m for 4 m/s (Figure 2B), 3.7 m for 6 m/s (Figure 2C), and 5.3 m for 8 m/s (Figure 2D). While density peaks at further distances as velocity increases, a negative correlation is revealed between player velocity (integers from 1 to 8 m/s) and peak commitment probabilities (ρ = -0.80 for *t* = 2 s), and between ball time-to-point (whole second integers from 1 to 4 s) and peak commitment probabilities (ρ = -1 for velocity = 4 m/s). At higher velocities, the probability that a player will commit to a contest decreases as the relative angle increases. For a velocity of 8 m/s or greater, player’s exhibit minimal influence on space in the negative y- axis (i.e., behind their direction of displacement direction). As velocity increases, we also note that the shape of a player’s commitment inverts.

### Decisions and Alternatives

**FIGURE 3:**
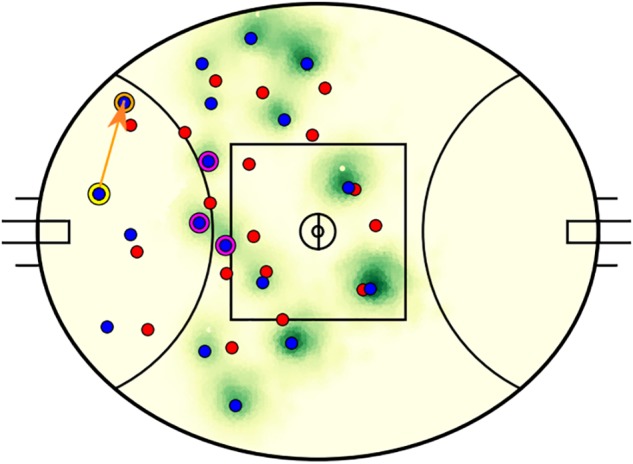
An example output of the decision-making model. The attacking team players are plotted in blue and their opponents in red. The kicker (circled in yellow) executed a pass along the orange line to the receiver (circled in orange). The model identified three higher valued passes (to players circled in magenta) toward the middle of the field that are within a 60 m radius of the kicker. The intensity of green correlates to the expected outcome of passes to each field position.

**FIGURE 4:**
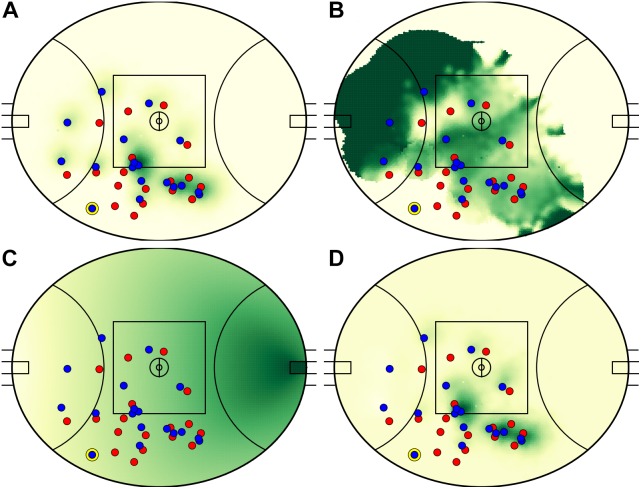
Team influence **(A)**, dominance **(B)**, field equity **(C)**, and resultant expected outcomes **(D)** relative to the player in possession (circled in yellow, toward the lower boundary). High value space is represented as darker green regions. Team influence measures the spatial influence of the attacking team (whose players are in blue), while dominance measures their spatial ownership relative to the opposition (whose players are in red). All values are calculated relative to the player in possession. When complete, the model presented in this paper identifies two high value areas toward the center square, both viable passing options (see **D**).

A total of 2935 passes matched the selection criteria across 60 matches (48.9 ± 14.7 kicks per match). An example decision-making output is visualized in Figure 3. In this example, the kicker passes to a teammate positioned toward the boundary line in the defensive 50 m region, while the model identified three higher value passes to teammates positioned toward the center of the field. Figure 4 presents the components that constitute EO calculations. Summarized characteristics of decisions and alternatives are presented in Table 2. The mean of all analyzed variables was lower for decisions compared to alternatives and all differences were statistically significant (refer to Table 2).

**Table 2:** Mean values for decision-making variables between decisions and alternatives.

Variable	Decisions	Alternatives
Dominance	0.66 ± 0.23	0.75 ± 0.23
Influence	0.51 ± 0.27	0.63 ± 0.31
Risk	0.33 ± 0.19	0.47 ± 0.21
Expected outcome	0.34 ± 0.46	2.11 ± 1.41
Decision value	0.13 ± 0.42	0.78 ± 0.24
Distance	25.0 ± 11.8	42.7 ± 17.8


*Values are presented as Mean ± SD and all differences are statistically significant.*

A very weak correlation was noted between vertical displacement from center and DV of decisions (ρ = 0.06). Horizontal displacement from the attacking team’s goal is positively correlated with DV (ρ = 0.56).

**FIGURE 5:**
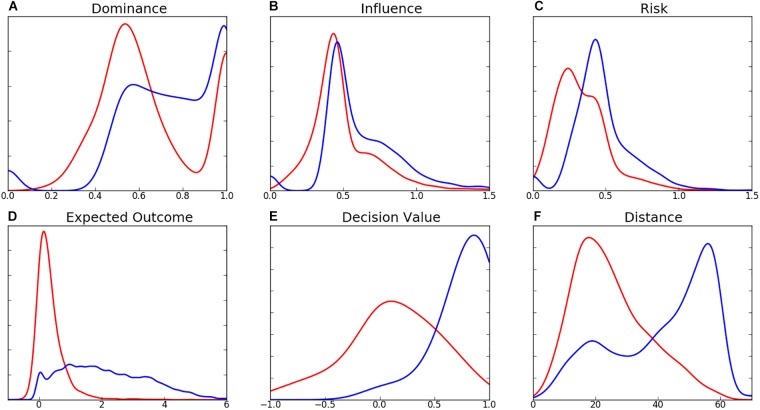
Distribution of **(A)** Dominance, **(B)** Influence, **(C)** Risk, **(D)** Expected Outcome, **(E)** Decision Value, and **(F)** Distance (m) for decisions (red) and alternatives (blue).

The distributions of decision-making characteristics are presented in Figure 5. The distribution of dominance (Figure 5A) is bimodal, with peak density for decisions at DOM = 0.54 and a local maximum at DOM = 1.0. This global peak at 0.54 represents a contest between two teams that slightly favors the attacker, while the local maximum at 1.0 represents a kick to an area of absolute dominance. The distribution of alternatives is similarly bimodal, with a greater negative skew and density around absolute dominance. Influence of decisions (Figure 5B) reveals peak density at INF = 0.43, which is comparable to the average peak density of player commitment models (Figure 2). Density for risk peaks at 0.25 (Figure 5C). The shape of the distributions of EO for decisions and alternatives are different, with decisions exhibiting peak density at EO = 0.14 (Figure 5D), and minimal density is noted at EO > 2, while alternatives are noted as having a greater range of EO values, with no notable density peak. DV follows a relatively normal distribution for decisions (Figure 5E) and distributions of kicking distance (Figure 5F) exhibit opposite skews (decisions are positively skewed, while alternatives negatively). Density of kicking distance for decisions is highest at 17.3 m, marginally longer than the 15 m minimum distance required for a legal mark. Small density peaks at 0.0 are observed for the dominance, influence, and risk of alternatives.

### Team-Level Characteristics

**FIGURE 6:**
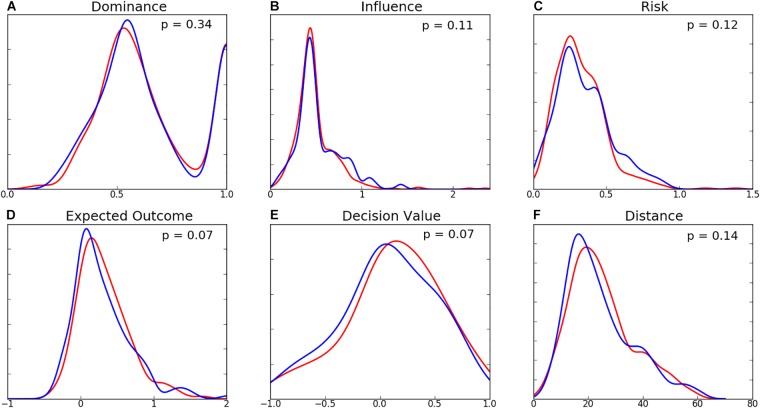
Distribution of team-level **(A)** Dominance, **(B)** Influence, **(C)** Risk, **(D)** Expected Outcome, **(E)** Decision Value, and **(F)** Distance (m) for Team A (blue) and Team B (red). Associated *p*-values (computed using the Mann-Whitney *U*-test) are presented for each variable.

**FIGURE 7:**
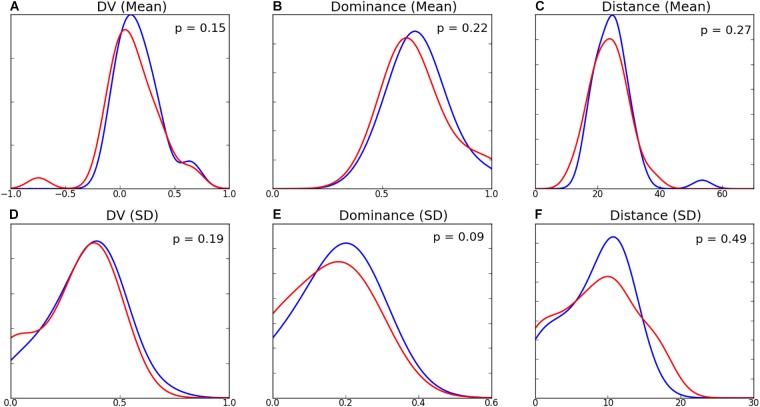
Within-team distributions for decision-making for Team A (blue) and Team B (red). Top row are the distributions for the within-team player means of **(A)** Decision Value, **(B)** Dominance, and **(C)** Distance (m), and the bottom row are the within-team player standard deviations of **(D)** Decision Value, **(E)** Dominance, and **(F)** Distance (m).

The distributions of passing characteristics for two teams are presented in Figure 6 and the summary statistics in Table 3. There was minimal difference in the dominance, influence, risk, and distance of decisions between the two teams. The mean EO and DV for Team B are higher than those of Team A, however no differences were found to be statistically significant. While the shape of variable distributions is similar for both teams, it is noted that Team B exhibits a greater negative skew for EO, DV, and distance variables. Distributions of mean decision-making characteristics for players amongst both teams were found to be similar (Figure 7). While the differences between player-level standard deviations were not found to be statistically significant, the distributions for dominance and distance variance display visual differences.

**Table 3:** Mean values for decision-making variables between Team A and Team B. *p*-values for differences are presented in Figure 5.

Variable	Team A	Team B
Dominance	0.66 ± 0.23	0.66 ± 0.23
Influence	0.52 ± 0.24	0.49 ± 0.24
Risk	0.34 ± 0.19	0.32 ± 0.17
Expected outcome	0.29 ± 0.39	0.34 ± 0.42
Decision value	0.08 ± 0.42	0.13 ± 0.43
Distance	24.3 ± 12.0	24.9 ± 11.6


## Discussion

This study demonstrates a method for measuring characteristics of player pass decision-making in invasion team sports. Previous studies of player decisions have measured decisions relative to some current measure of possession expectation (e.g., Cervone et al., 2014), rather than relative to the value of alternative passes that were presented. While the former approach assigns value to a specific kick, relative measures of decision-making assign value to individual decisions. Similar to the distinction between player accuracy and shot difficulty (e.g., Chang et al., 2014), assigning value to player decision-making presents greater insights into individual player performance. The adoption of decision-making evaluation in combination with measurements of accuracy and risk would allow for targeted coaching and recruitment, as well as defining categories of player tactical behavior.

The methodology presented in this study has practical applications in performance analysis. Understanding the components of player behavior that contribute to their overall performance can be used by sporting teams to target coaching or recruitment practices. For example, understanding if a player has poor execution but good decision-making, or vice versa, provides meaningful insights into said players individual performance. This concept could be explored further in future work by analyzing player decisions in response to match events. It is likely that a player’s decision-making abilities vary based on external stimulus such as opposition pressure. Understanding these components of player performance allows for more specific recruitment of player types. For example, should teams tend toward a style of decision-making (i.e., risk aversion), the quantification of player decisions would allow for more informed recruitment.

A major component of the decision-making modeling were player motion models, fit on the weighted distributions of player commitment to contests. While previous studies have developed probabilistic motion models with arbitrary bounds (Fernandez and Bornn, 2018) or from a player’s observed displacements (Brefeld et al., 2018), the commitment modeling approach demonstrated in this study fits player behavior with consideration of movement context, representing a new approach to the measurement of a player’s spatial influence. Furthermore, the models are parameterized through the fitting of density in four dimensions (with consideration of a player’s velocity, time and x- and y- co-ordinates), presenting a continuous representation of player commitment. A notable finding of the motion models is that commitment peaks are of lower density for higher velocity and time values. That is, players are overall less likely to commit to an upcoming contest if the ball is further away (hence, a high time-to-point) or if they are moving at high velocities. This finding is logical and may be explained by a desire to simply corral an opponent or reposition for future involvements, rather than participate in the immediate transaction. As with alternative motion models, we found that a player’s influence in the negative y-axis (i.e., behind them) degrades as their speed increases. While models fit on player commitments more realistically measure their likelihood to occupy future space, the models only consider a player’s position and momentum, not teammate locations. A player’s participation in a contest logically has some dependence on the position of their teammates, hence attempts to incorporate may produce more realistic models.

A key finding in this study are the novel insights into the decision-making and passing tendencies of Australian Rules footballers. Previous studies have identified the importance of kicking in the AFL (Stewart et al., 2007; Robertson et al., 2016) but there has been minimal work into describing the kicking landscape at elite levels at a transactional level (e.g., distance, level of pressure), despite studies on the biomechanics of kicking in Australian Rules football (e.g., Ball, 2008a,b). This study found that kicks resulting in a mark are most commonly short, with a density peak at 17.3 m (mean = 25 m), marginally longer than the minimum distance required for a legal mark. This could be the result of tactical behavior, or indicative of the ease in which close options can be identified due to lower visual obstruction. Furthermore, successful marks are most often to players in one on one contests or to players who are completely open (as suggested by the bimodal distribution of passing dominance and the density peaks of risk), which may be indicative of risk aversion, however more research is required to understand individual player behavior. These insights into the passing behavior of Australian footballers has practical applications in training practices. A recent study by Robertson et al. (2019) suggested analyzing passing constraints in the AFL to ensure training conditions represent those experienced in a match. The metrics developed in this study are continuous and measured from positional data, hence may provide more objective results than the manually collected passing constraints in Robertson et al. (2019). Understanding the spatial characteristics of passes may allow for coaching staff to prescribe training drills that reflect the spatial pressure experienced by players during match conditions.

In contrast to player decisions, the optimal alternative passes that were identified by the model presented in this study were long distance kicks, less frequently to unmarked individuals. While the distribution of dominance was similarly bimodal for alternatives, the peak at absolute dominance (DOM = 1.0) was less intense than for decisions. The higher density for passes to areas of dominance between 0.5 (a 50/50 contest) and 1.0 suggests kicks to areas in which multiple teammates have an opportunity to receive the ball. This is reinforced by the distribution of influence for alternatives (Figure 5B) where more density is noted for influence above 0.5 compared to decisions. Long-range passes having higher associated values (EO and DV) is logical due to the inclusion of AFL field equity, in which the value of space increases as the distance and angle to the goalposts decreases (Jackson, 2016; Figure 4C). The contrast in distances between decisions and alternatives (Figure 5F) could be due to several factors, such as a difficulty for players to identify long-range options (due to visual obstruction and lower decision-making time, for example) or an underestimation of kicking accuracy by the model. Due to the unavailability of precision ball tracking in AFL, this study used an arbitrary measurement of kicking accuracy. Should more detailed transactional data or LPS ball tracking become available, it is believed that kicking accuracy could be modeled from empirical data. The density peaks at values toward 0 for dominance, influence, and risk can be explained by situations in which all passing options are positioned in areas of negative field equity (e.g., field formations in the defensive 50 m area), resulting in an optimal decision being a kick to an area of no spatial dominance (hence, no negative associated equity). This is a common problem with models that use equity-based rewards, where moving the ball backwards is often associated with a reduction in equity. A further limitation of the equity component used in this study is its lack of consideration for teammate and opponent positions. This metric predates the widespread availability of player tracking information in the AFL, hence only considered possession location and source in its computation (O’Shaughnessy, 2006). An updated metric that considers player locations may improve the accuracy of this decision-making model.

Team level analysis revealed that the less successful team in the 2017/2018 season had higher average DV than the more successful team. Furthermore, while within-team distribution of player averages were similar, the player variance of DV was more positively skewed for the less successful team. Of particular interest is the finding that the less successful team executed passes of higher value, potentially suggestive of a difference in playing styles. Future research into player and team-level decision-making, should consider contextual information such as match conditions, score deficits, and tactical styles.

The tactical behavior of teams has been explored via network behavior, in which the connectivity between players is quantified via the frequency of passing between them in soccer (for examples, see Pena and Touchette, 2012; Gonçalves et al., 2017) and basketball (Fewell et al., 2012). While some of these studies have utilized spatiotemporal datasets (e.g., Gonçalves et al., 2017), this has not been used to measure differences in the spatial characteristics of passes between players. Furthermore, it is possible that the decision-making of links in said passing networks varies – that is, do some players have a tendency to create passing links to teammates of quantitatively lower valued decisions? The quantitative measurement of these links (in terms of DV and EO) may yield insights into the tactical behaviors of teams, as well as the decision-making of individual players. This work could be used to measure a player’s perceived skill based on their teammates willingness to execute lower valued or riskier passes to said player.

Despite these differences in the mean and standard deviation of team-level metrics, we note that the differences were not meaningfully different. Compared to the league-wide averages, the greatest differences experienced by either team were of Team A’s DV and EO. Given that the decision-making model is developed from league-wide averages, this may suggest that Team A executes passes at a level above the league average. The reward component is fit on the average equity gain, given field location and pressure, hence it is possible that individual team equity gains may have significant variation. Future research into the decision-making of Australian footballers should consider differences in outcomes to identify if there is a difference in the execution of passes between teams. That is, do certain teams outperform the mathematical averages of this decision-making model? At an individual level, this analysis could be used to identify players who are executing passes above the average of their cohort.

## Conclusion

This work represents the beginning of ongoing research into player decision-making in the AFL. The decoupling of player decision-making from overall player performance allows for a more precise understanding of player ability that has applications in coaching and scouting. Underlying the decision-making model is a player motion model fit on the combined distributions of relative contest locations that were committed to, and those that were not. The resulting motion model quantifies the probability that a player would commit to a contest, given their velocity, displacement direction, and past behaviors. It was found that player commitment decreases as a function of velocity and available time, offering insights into the commitment behavior of players. Analysis of passes revealed that players typically execute short kicks that are most commonly to teammates in one-on-one or unmarked situations, resulting in a bimodal distribution of passing dominance. Conversely, the mathematical model presented in this paper identifies long-range options as having higher expected value, given the inclusion of field equity which rewards possession closer to the goalposts. This mismatch indecisions could be due to the ease in which short-range options can be identified and executed compared to long-range options. Differences in decision-making variables between two analyzed teams suggests a need for expanded datasets and research into player decision-making with consideration of match context.

## Ethics Statement

Players provided their data and written and informed consent to the commercial provider as part of their collective bargaining agreement. Ethical approval for this study was granted by Victoria University’s Human Research Ethics Committee (VU HREC 24514).

## Author Contributions

BS, KJ, and SR: conceptualization. BS and KJ: methodology. TB: data extraction. BS and TB: data cleaning. BS: data analysis and visualization. BS and SR: writing. BS, SR, KJ, and TB: drafting.

## Conflict of Interest Statement

KJ and TB were employed by Champion Data. The remaining authors declare that the research was conducted in the absence of any commercial or financial relationships that could be construed as a potential conflict of interest.
